# Obstetric Cardiac Arrest: A Case Report

**DOI:** 10.7759/cureus.67847

**Published:** 2024-08-26

**Authors:** Nikolina Madjer, Daniel Sherlock, Andrii Labchuk, Katarzyna Mikrut

**Affiliations:** 1 Internal Medicine, Advocate Lutheran General Hospital, Park Ridge, USA; 2 Cardiology, Advocate Lutheran General Hospital, Park Ridge, USA

**Keywords:** inari, obstetric and orthopedic trauma, emergency cesarean section, ekos catheter, massive pulmonary embolism, in hospital cardiac arrest, emergency obstetric care

## Abstract

Cardiac arrest during pregnancy does not occur infrequently and is influenced by obstetric and non-obstetric factors. The patient described in this case report is a pregnant woman who suffered a leg injury that required urgent surgical repair. Moments prior to that procedure, the fetus experienced extreme bradycardia on fetal heart tone monitoring. An emergent cesarean section was performed, which was followed by the patient suffering cardiac arrest secondary to an acutely provoked pulmonary embolism. The patient underwent mechanical thrombectomy followed by EkoSonic endovascular system (EKOS) therapy, which was then complicated by a subcapsular hematoma. The patient ultimately had an inferior vena cava (IVC) filter placed, was started on oral anticoagulation, and eventually recovered with discharge to her home with her newborn infant. This report aims to discuss this critical case of obstetric cardiac arrest, detailing the emergent response, clinical management, challenges faced during resuscitation, and subsequent outcomes. Through this report, we seek to contribute to the growing body of knowledge on effectively managing cardiac emergencies in pregnancy, emphasizing interdisciplinary coordination and tailored interventions to enhance survival and recovery in this high-risk group.

## Introduction

A critical event, such as a cardiac arrest during pregnancy, presents unique challenges and demands prompt, specialized care to ensure the best possible outcomes for both the woman and the child. While rare, the rate of obstetric cardiac arrest calculated in recent years has been higher than previous estimates, necessitating further research into this area of medicine. This emergency requires a multidisciplinary approach involving obstetrics, cardiology, anesthesiology, critical care, and emergency medicine.

The physiological changes that occur in women during pregnancy include increased cardiac output, decreased systemic vascular resistance, and compression of the inferior vena cava by the gravid uterus. Cardiovascular disease (CVD) is known to be a significant cause of mortality and morbidity in pregnant women. Arrhythmias are the most common presenting complaints during pregnancy, but most have a benign course [1]. These changes in maternal physiology and cardiac risk factors have spurred organizations such as the Society for Obstetric Anesthesia and Perinatology (SOAP) and the American Heart Association (AHA) to provide guidelines and recommendations for the management of cardiac arrest in pregnancy [2]. Despite these formal recommendations and increased awareness of this high-risk population, outcomes of maternal cardiac arrest during pregnancy are variable. It is generally accepted that cesarean sections within four minutes of cardiac arrest yield the highest rates of maternal survival [3]. Some studies have even concluded that high-risk CVD patients, such as those with adult congenital heart disease, history of cardiac arrest, connective tissue disease with aortopathy, ischemic cardiomyopathy, non-ischemic cardiomyopathy, or valve disease, have more favorable outcomes with main operating theater deliveries [4]. Pulmonary embolus (PE) is a well-known CVD complication of pregnancy, with an incidence of approximately 1 in 1,000 pregnancies, but the options for treatment are limited and the mortality rate is significantly higher (approximately 3%) than in non-pregnant women [5]. Standard treatments, such as thrombolysis and embolectomy, are often contraindicated in pregnancy, thus treatment options include delivery of the fetus or placing the patient on extracorporeal membrane oxygenation (ECMO) [6].

## Case presentation

**Figure 1:**
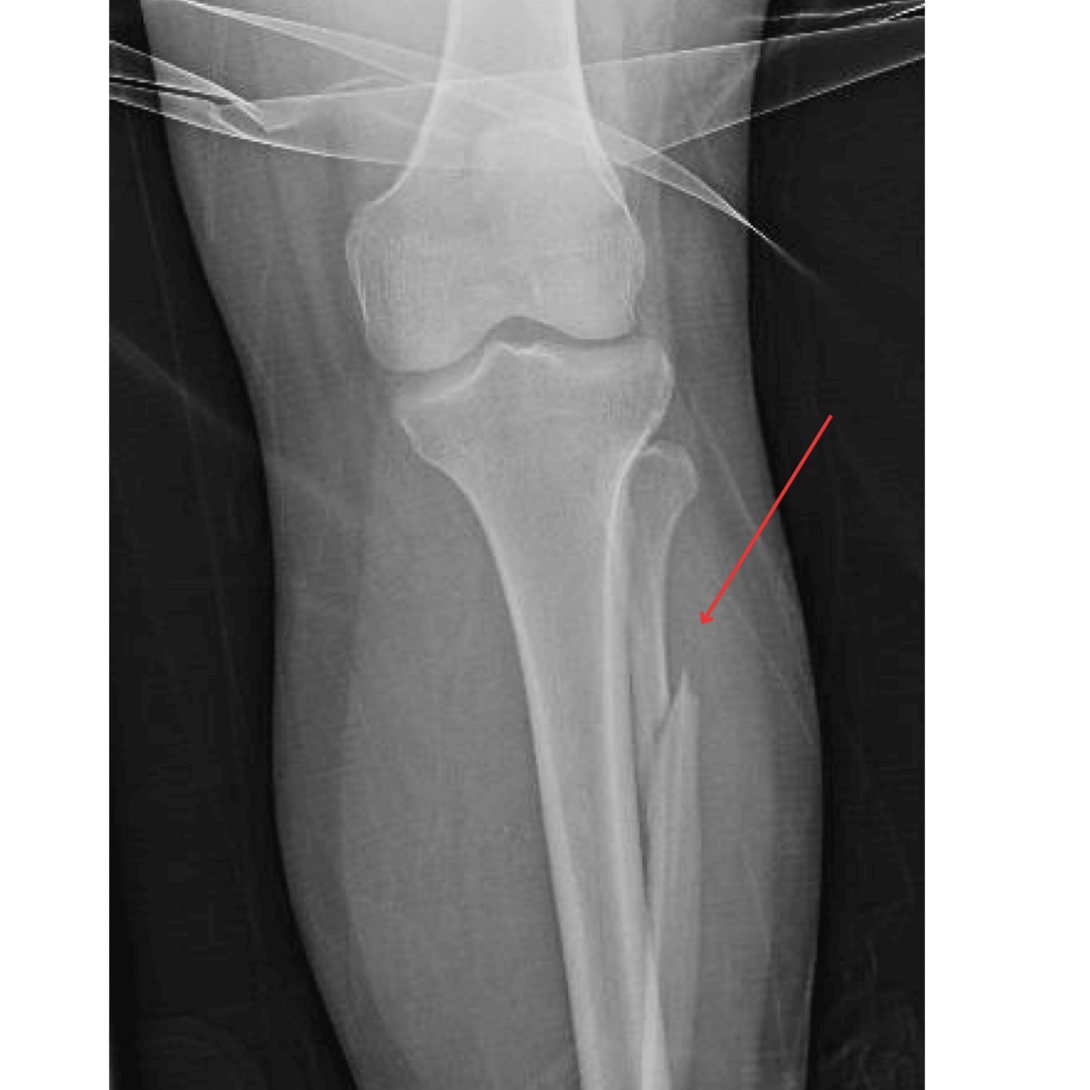
X-ray of the proximal left fibula fracture

**Figure 2:**
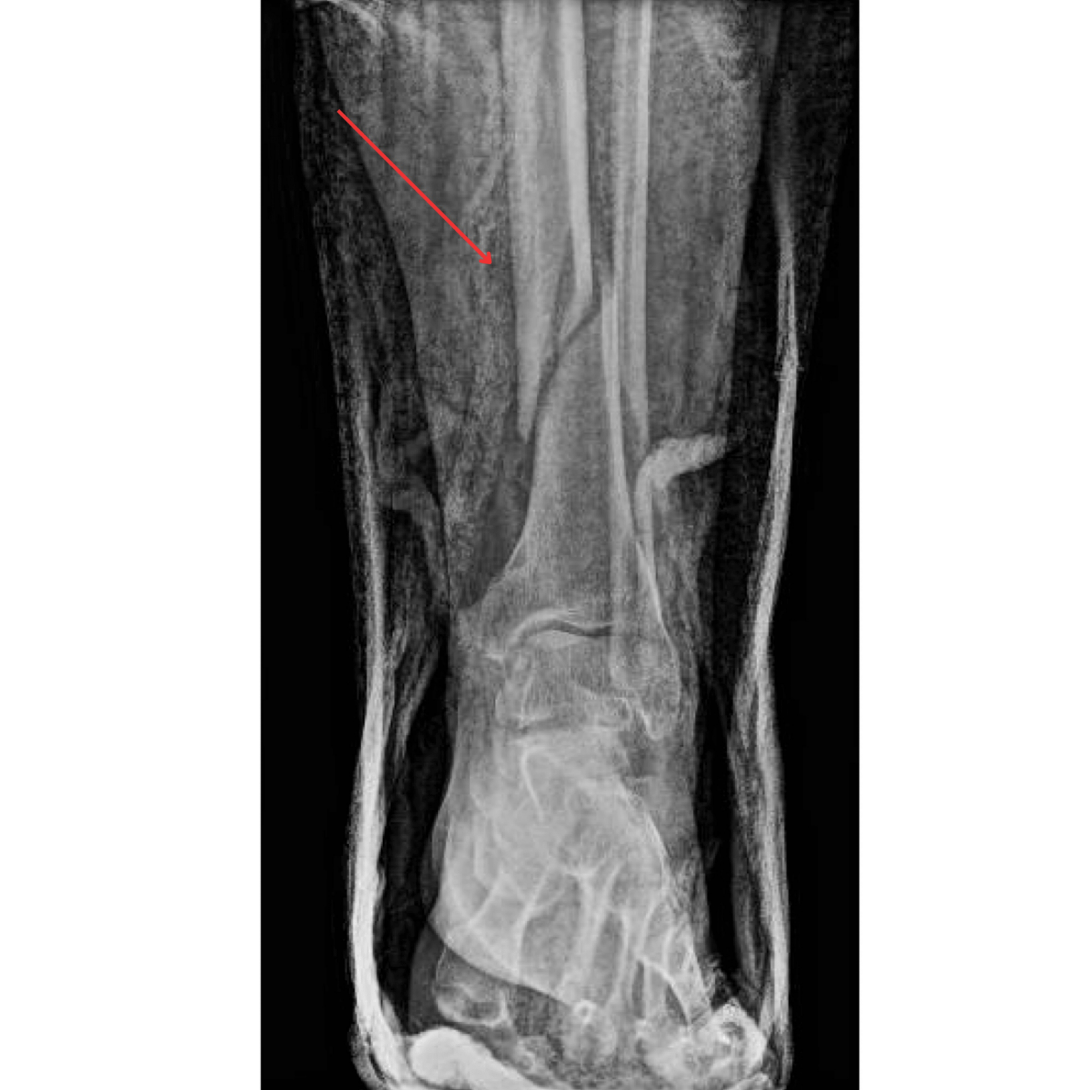
X-ray of the distal left tibia fracture

This patient was a 33-year-old female G1P0 with a past medical history significant for female infertility, who initially presented to an outside hospital (OSH) at 34 weeks pregnant with the chief complaint of ankle pain. The patient had reported that while walking her dog at her local dog park, two large dogs collided with her legs from behind, causing her to lose balance and fall backward. Immediately after that event, the patient had severe ankle pain and concerns about decreased fetal movement, prompting her to visit the emergency department (ED) at the OSH. In the ED, the obstetrics team confirmed a strong fetal heartbeat. Shortly thereafter, the patient underwent an X-ray of the left tibia, fibula, and ankle, which revealed acute moderately displaced fractures of the distal left tibia and proximal left fibula (Figures 1, 2). Orthopedic Surgery recommended surgical fixation. Given that the patient achieved conception via in vitro fertilization (IVF), the patient was considered a high-risk pregnancy and would require closer monitoring during an invasive operative procedure by Maternal-Fetal Medicine (MFM). The patient was transferred to a tertiary care center where orthopedic surgery and MFM could work simultaneously during the tibia-fibula repair. 

As the patient was being wheeled down to the operating room (OR), she had vasovagal-like episodes prior to the administration of anesthesia. At that time, the anesthesiologist requested fetal monitoring. The MFM team also arrived at the OR to consent the patient for a cesarean section should it be needed during the surgery. The patient continued to waver in and out of consciousness. Then, fetal heart tones (FHTs) fell into rates of fifties with absent variability, which was confirmed by bedside ultrasound and Doppler. In approximately 60 seconds, an emergent c-section was performed, which was followed by maternal pulseless electrical activity (PEA) arrest. The patient was emergently intubated and chest compressions were started. Return of spontaneous circulation (ROSC) was achieved in 10 minutes. The estimated blood loss during the c-section was approximately one liter, prompting the start of the massive transfusion protocol in which the patient received one unit of fresh frozen plasma, one unit of platelets, one unit of packed red blood cells, and one unit of cryoprecipitate. She was also administered one gram of tranexamic acid, misoprostol per rectum, and intramuscular methergine.

**Figure 3:**
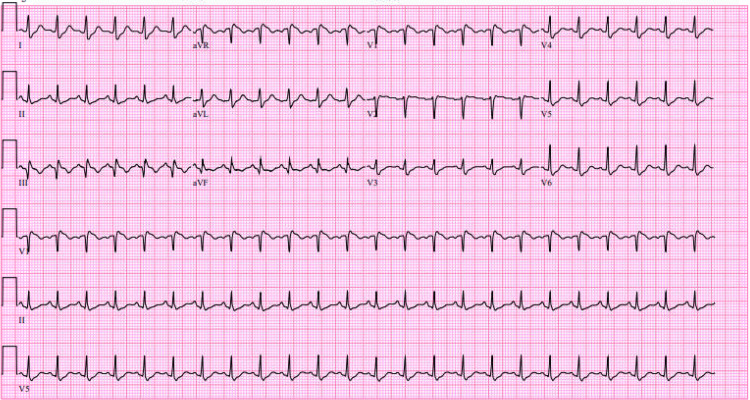
EKG demonstrating the S1Q3T3 pattern EKG: electrocardiogram S1Q3T3 denotes a negative "S" wave in lead I, a deep "Q" wave in lead III, and an inverted "T" wave in lead III.

**Figure 4:**
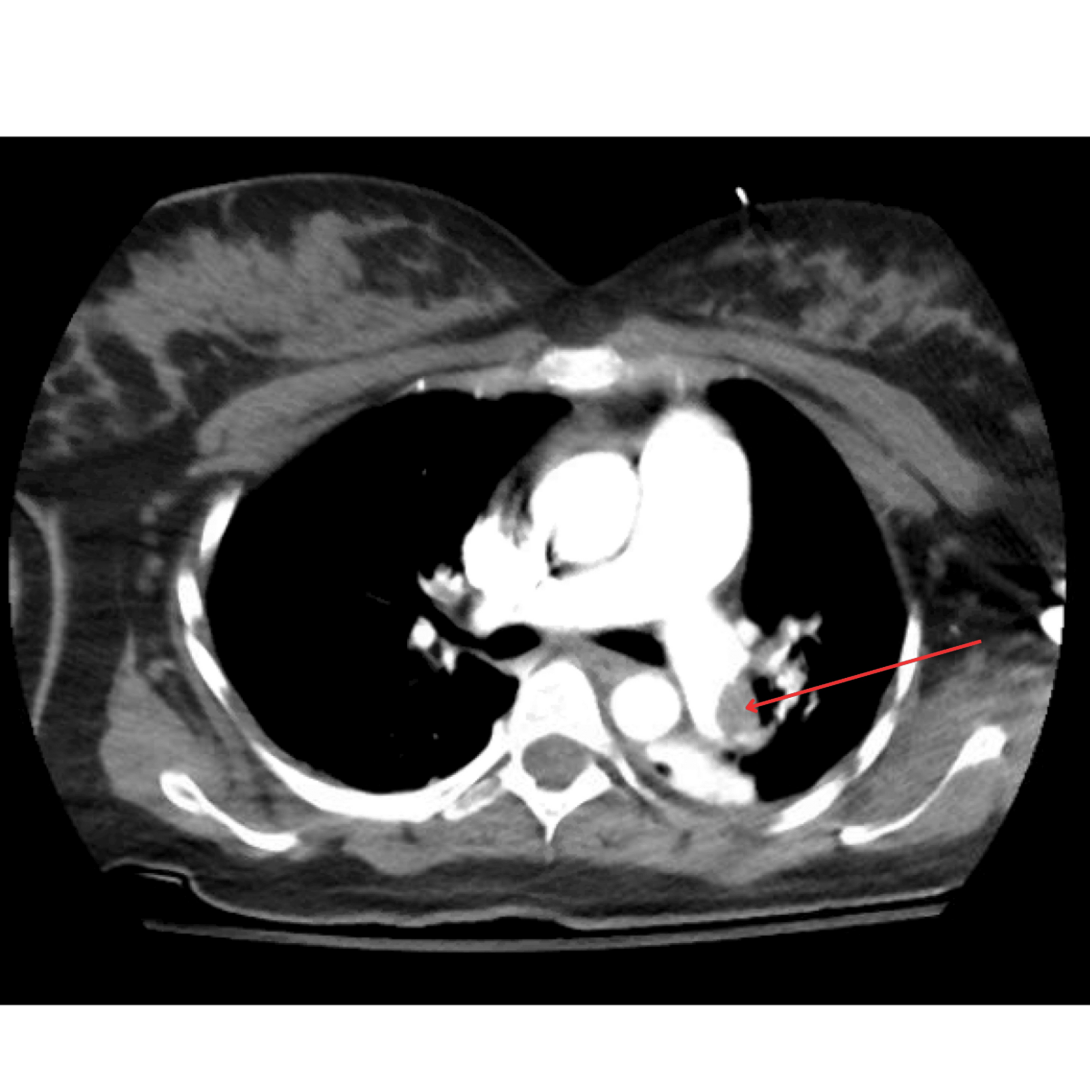
CTA chest pulmonary embolism CTA: computed tomography angiography Demonstrating embolus in the left distal main pulmonary artery

**Figure 5:**
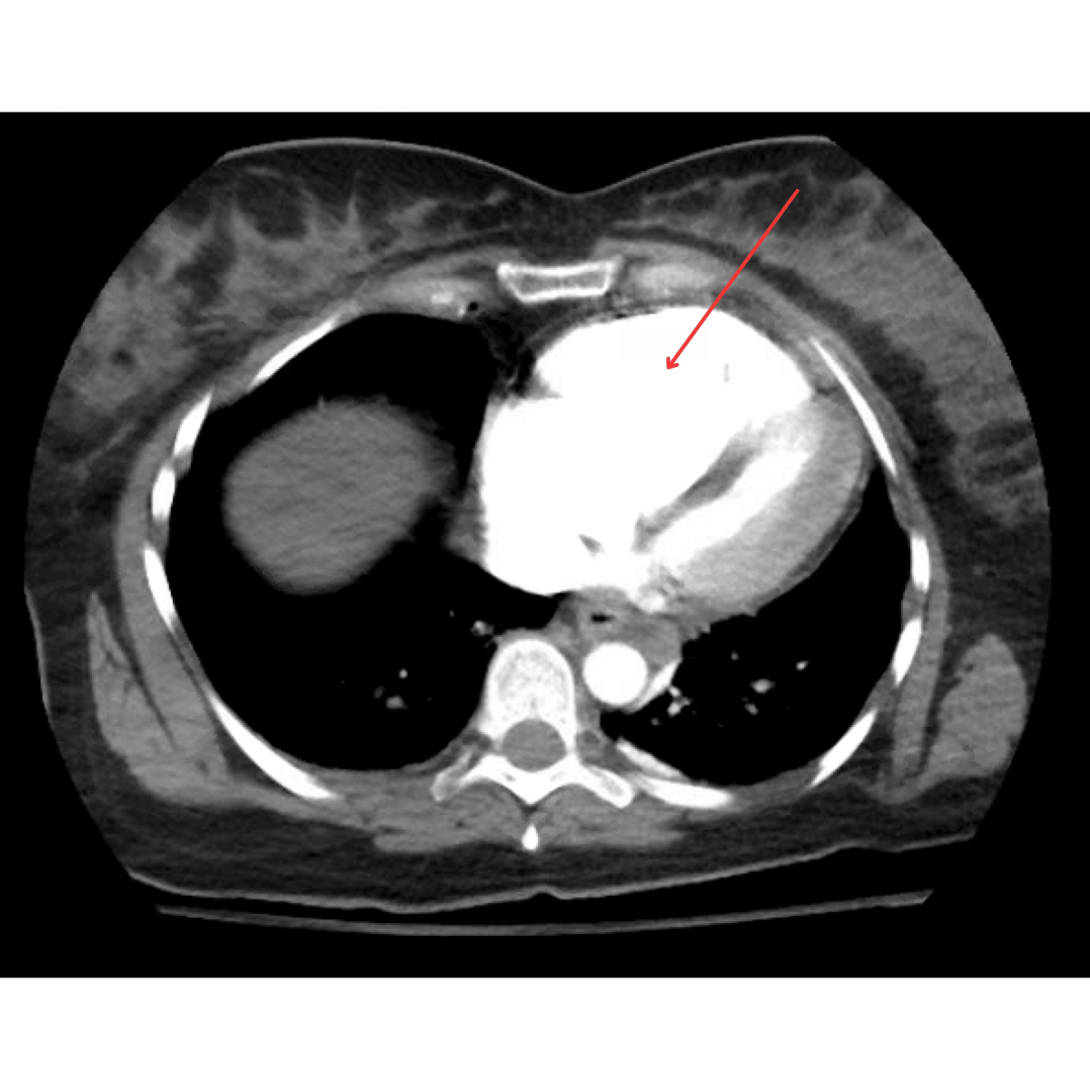
CTA chest pulmonary embolism CTA: computed tomography angiography The right ventricle to left ventricle ratio was measured as greater than 1.0, with findings suggestive of right heart strain in the setting of identified pulmonary emboli.

Following ROSC, the electrocardiogram (EKG) showed sinus tachycardia with an S1Q3T3 pattern (Figure 3). With those findings, along with heightened risk factors for a hypercoagulable state, there was concern for an embolic event as the precipitating cause of the patient’s cardiac arrest. CT angiography (CTA) chest pulmonary embolism revealed large-volume acute pulmonary emboli in the distal main, lobar segmental, and subsegmental pulmonary arteries of both lungs, with evidence of significant right heart strain. The embolic burden was most notable in the pulmonary arteries of the bilateral lower lobes. The right ventricle (RV) to left ventricle (LV) ratio was greater than 1.0, and contrast was from the right atrium into the IVC (Figures 4, 5). Transthoracic echocardiogram (TTE) also confirmed right heart strain with ventricular septal flattening in systole suggestive of RV pressure overload, severely dilated RV cavity size, and reduced systolic dysfunction.

The patient was then taken to the cardiac catheterization laboratory for an emergent Inari catheter-directed thrombectomy. There was retrieval of a large amount of clots with 24F and 16F catheters from the right and left lungs. Due to a small residual thrombus in the right pulmonary artery and a large residual thrombus in the left pulmonary artery, bilateral EkoSonic endovascular system (EKOS) catheters were inserted via the right internal jugular vein and the patient was started on tissue plasminogen activator (tPA) and a heparin infusion.

**Figure 6:**
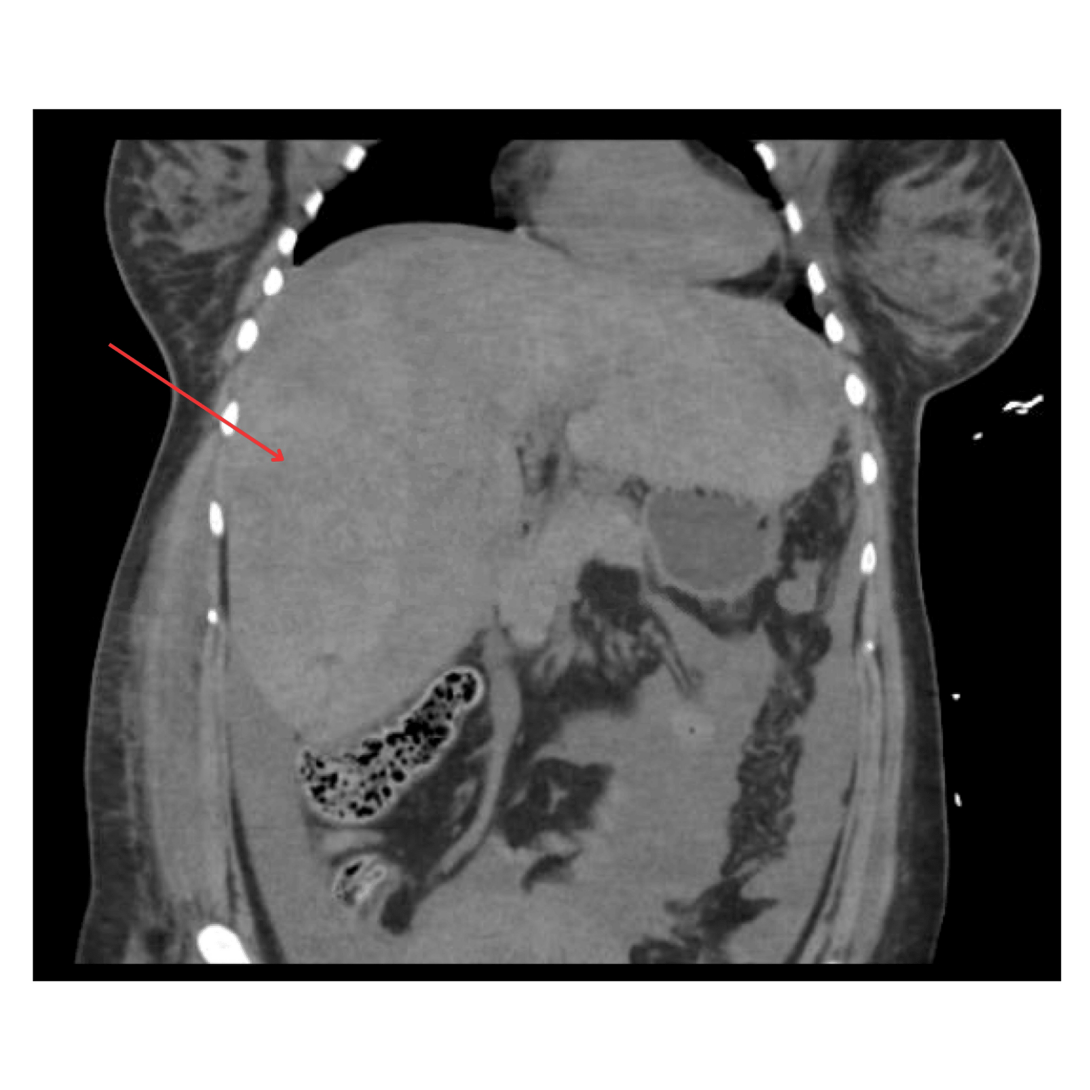
CT abdomen CT: computed tomography (CT) Large subcapsular hematoma seen along the anterior margin of the entire liver. This measures approximately 6.4cm in the greatest thickness along the right hepatic lobe.

By the next day, the patient was extubated and alert while continuing EKOS therapy. She was expressing severe abdominal pain, particularly in the right upper quadrant, which correlated with her physical exam showing an enlarged abdomen measuring 107 cm. A bedside ultrasound revealed free fluid in the abdomen. At that point, tPA and heparin were discontinued. Laboratory testing showed a drop in hemoglobin from 10.8 g/dL to 6.4 g/dL. A repeated CT abdomen pelvis revealed a significant size increase of a large subcapsular liver hematoma with brisk active hemorrhage and moderate volume hemoperitoneum. The hematoma was also causing a mass effect on the underlying liver (Figure 6). Interventional radiology (IR) embolization was attempted, but no bleeding vessels were visualized with hepatic angiography, likely due to parenchymal compression by the subcapsular hematoma. General surgery was consulted at that time, who evaluated the patient and decided that given there was no active bleeding seen during the IR procedure, and her physical exam was stable with a tender but soft abdomen; there was no indication for an acute surgical procedure. They also commented that the bleeding would eventually cease on its own because it was likely venous. Their final recommendations were to closely monitor her hemoglobin levels and do serial abdominal exams to monitor for acute abdominal compartment syndrome. Thankfully, the patient never experienced this.

Given that all anticoagulation had to be discontinued, an inferior vena cava (IVC) filter was inserted. She remained off anticoagulants for two days. At that time, her platelet count dropped (225 K/mcL to 63 K/mcL in 24 hours) so hematology was consulted due to concern for heparin-induced thrombocytopenia and thrombosis (HITT). The patient was placed on argatroban while waiting for platelet factor 4 testing, which eventually resulted negative. For that reason, she was transitioned to enoxaparin with no further episodes of bleeding or thrombocytopenia.

The patient underwent an open reduction and internal fixation of her tibia and fibula fractures approximately nine days after the cardiac arrest and tolerated the procedure well. Given the patient’s desire to breastfeed her newborn son, hematology planned to discharge her on an enoxaparin bridge to warfarin, which was deemed safe for breastfeeding. Today, the patient and her baby are recovering well with close follow-up with all consultant groups in the outpatient setting.

## Discussion

This case report illustrates the complex interplay of trauma, critical care, obstetrics, and thromboembolic complications in a high-risk obstetric patient. The patient, a 33-year-old female with a history of infertility treated with IVF, presented with a traumatic injury leading to orthopedic surgery, which was further complicated by a peripartum cardiopulmonary arrest secondary to a massive pulmonary embolism. The incidence of thromboembolism, including deep vein thrombosis (DVT) and pulmonary embolism (PE), is known to be higher during pregnancy, with thromboembolic disease being the leading cause of maternal mortality in the developed world [7]. Risk factors for thromboembolism in pregnancy include advanced maternal age, obesity, smoking, immobilization, and thrombophilia [8]. Moreover, the antepartum risk of VTE for IVF pregnancies is doubled compared to spontaneous conceptions [9]. This patient’s fall and subsequent tibia and fibula fractures necessitated surgical intervention. Unfortunately, this patient's surgical fracture fixation was delayed, which is another known risk factor for thrombosis in the general public [10].

While orthopedic surgery is crucial in the management of such fractures, it poses a challenge in the setting of pregnancy due to the need for fetal monitoring and the potential for complications like fetal distress and preterm labor. Regardless, the decision to transfer the patient to a tertiary care center for combined orthopedic and MFM management was prudent in providing specialized care. The vasovagal episodes experienced by the patient prior to anesthesia raised concerns for an underlying cardiovascular event, which later manifested as a severe PE.

PE in pregnancy can present with nonspecific symptoms and is often underdiagnosed due to overlapping symptoms with normal pregnancy [11]. The EKG findings of the S1Q3T3 pattern, along with the echocardiographic evidence of right heart strain, were indicative of a significant PE, which was confirmed by the CTA chest. EKG findings in acute pulmonary embolism are frequently normal (18-29% of cases), as sensitivity and specificity of the S1Q3T3 pattern for acute pulmonary embolism is relatively low. However, when this pattern is present, it can often act as a sign for further investigation with clinical suspicion [12].

Catheter-directed therapies (CDT) are emerging as effective treatments for acute massive and submassive PE [13]. The use of the Inari catheter system and EKOS therapy in this case facilitated the removal of large-volume clots, which contributed to the patient’s survival. However, the subsequent development of a subcapsular liver hematoma highlights the delicate balance between thrombolysis and the risk of hemorrhage [13].

The management of anticoagulation in the context of significant hemorrhage requires careful consideration. The patient’s drop in hemoglobin and hemodynamic instability necessitated the temporary cessation of anticoagulation therapy, followed by an IVC filter placement to prevent further pulmonary embolic events [14,15].

## Conclusions

This case highlights the need for a vigilant and proactive approach to the care of pregnant patients with multiple risk factors for thromboembolism. It underscores the importance of interdisciplinary collaboration in managing complex cases that span across different specialties and require a coordinated effort to optimize patient outcomes. The successful management of this patient, who experienced a cascade of events, including trauma, massive PE, cardiac arrest, emergent cesarean section, and hemorrhagic complications, demonstrates the resilience of modern medical care. The ability to rapidly diagnose and intervene in critical situations while navigating the delicate balance between thrombosis and bleeding risks is a testament to the expertise and dedication of the healthcare team involved.

Further research and collaboration among the various medical specialties discussed in this report are warranted to enhance our understanding of the optimal management strategies for similar high-risk obstetric cases. By sharing experiences and outcomes, we can continue to improve the care and outcomes of pregnant patients facing complex medical scenarios.
